# A synonymous change, p.Gly16Gly in *MECP2* Exon 1, causes a cryptic splice event in a Rett syndrome patient

**DOI:** 10.1186/1750-1172-8-108

**Published:** 2013-07-19

**Authors:** Taimoor I Sheikh, Kirti Mittal, Mary J Willis, John B Vincent

**Affiliations:** 1 Molecular Neuropsychiatry & Development Lab, Campbell Family Mental Health Research Institute, Centre for Addiction & Mental Health, Toronto, Canada; 2 Institute of Medical Science, University of Toronto, Toronto, Canada; 3 Clinical Genetics, Naval Medical Center, San Diego, USA; 4 Department of Psychiatry, University of Toronto, Toronto, Canada

**Keywords:** Cryptic splice site, Synonymous mutation, *MECP2*, exon 1, Rett syndrome, Silent mutation, Frame-shift mutation

## Abstract

**Background:**

Mutations in *MECP2* are the main cause of Rett Syndrome. To date, no pathogenic synonymous *MECP2* mutation has yet been identified. Here, we investigated a *de novo* synonymous variant *c.48C>T* (*p.Gly16Gly*) identified in a girl presenting with a typical RTT phenotype.

**Methods:**

*In silico* analyses to predict the effects of sequence variation on mRNA splicing were employed, followed by sequencing and quantification of lymphocyte mRNAs from the subject for splice variants *MECP2_E1* and *MECP2_E2*.

**Results:**

Analysis of mRNA confirmed predictions that this synonymous mutation activates a splice-donor site at an early position in exon 1, leading to a deletion (*r.[=, 48_63del]*), codon frameshift and premature stop codon (p.Glu17Lysfs*16) for *MECP2_E1*. For *MECP2_E2*, the same premature splice site is used, but as this is located in the 5′untranslated region, no effect on the amino acid sequence is predicted. Quantitative analysis that specifically measured this cryptic splice variant also revealed a significant decrease in the quantity of the correct *MECP2_E1* transcript, which indicates that this is the etiologically significant mutation in this patient.

**Conclusion:**

These findings suggest that synonymous variants of *MECP2* as well as other known disease genes—and *de novo* variants in particular— should be re-evaluated for potential effects on splicing.

## Introduction

Rett syndrome (RTT; MIM#312750) is an X-linked neurological disorder which leads to gradual slowing of neurodevelopment in females. Clinical features and symptoms may include microcephaly, repetitive hand movement, scoliosis, constipation, excessive saliva, intellectual disability (ID), and typically little or no verbal skills.

**Figure 1:**
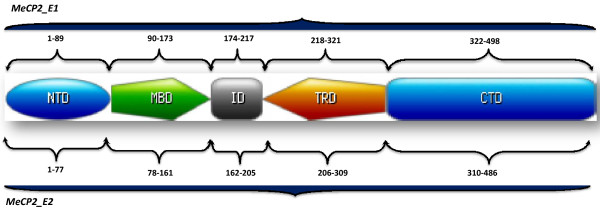
**MeCP2 Domain Structure: NTD: N-terminal domain, MBD: methyl-CpG DNA binding domain, ID: intervening domain, TRD: transcription repression domain and CTD is C-terminal domain.** Domain-wise distribution of amino acids of both isoforms of MECP2 protein. Amino acid counts for each domain of MeCP2_E1 was based on that for a previously described MeCP2_E2 domain structure [8].

Methyl-CpG binding protein 2 (MeCP2; MIM 300005) [1] located at Xq28, was identified as the gene responsible for RTT [2]. Initially, MeCP2 was identified due to its selective binding (via a methyl-CpG binding domain (MBD)) to DNA sequences that are methylated at cytosine in the dinucleotide CpG [3]. Subsequently, other highly conserved and functionally relevant domains, namely the transcriptional repression domain (TRD) and nuclear localization signal (NLS) were also identified [4,5] (Figure 1). Other functionally less well characterized domains include a C-terminal domain (CTD), which putatively binds to histone [6]. The MBD of MeCP2 does not only recognize methylated DNA, but can also bind unmethylated DNA. Additional unmethylated DNA binding sites are present between the MBD and TRD, and are known as the intervening domain (ID) (Figure 1) [7].

The canonical version of *MECP2* gene consists of 4 coding exons. The translation START site is in exon 2, and exon 1 and most of exon 2 are within the 5′untranslated region (UTR). However, a splice variant of *MECP2* was discovered later, namely *MECP2_E1*, in which exon 2 is spliced out and translation is initiated from a START codon in exon 1, thus resulting in a slightly larger protein with a different N-terminal [9,10] (Figure 1). The two isoforms are identical for the remainder of the protein, and both contain the MBD and TRD (Figure 1). Several studies suggested that MeCP2_E1 is the predominant isoform in brain tissues, with 10-fold higher expression [9,10]. *MECP2_E1* is not only transcribed at much higher levels in the brain than the canonical splice variant, *MECP2_E2*, but is also translated more efficiently. The presence of the upstream open reading frame appears to inhibit the efficient translation of MeCP2_E2 [10]. The N-terminal region encoded by *MECP2_E1* is highly conserved across all vertebrate groups, and is identical among many mammalian species [11].

There are currently over 250 known *MECP2* gene mutations that cause RTT [12]. Over 80% of patients with RTT have mutations in exons 3 or 4 of *MECP2*. The identification of disease-relevant mutations in exon 1 led to the likelihood that MeCP2_E1 is the etiologically relevant protein isoform for Rett [9,13,14], later confirmed by studies of mouse knockouts specific to isoform MeCP2_e2 [15], and has also led to the inclusion of exon 1 in diagnostic sequencing for Rett syndrome.

**Figure 2:**
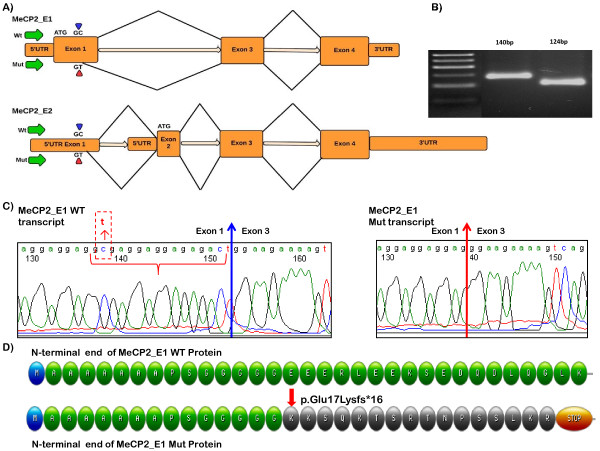
**Activation of cryptic splice site at c.48C>T in MECP2 exon 1: A) ideogrammatic representation of the genomic organization of *MECP2, *showing the alternative pre-mRNA splicing of wild type (WT) and mutant (Mut (NM_001110792: c.48C>T)) transcripts for both isoforms, MeCP2_E1 and MeCP2_E2; B) Agarose gel electrophoresis of reverse transcription-PCR product for WT (Lane 2, 140bp) and Mut (Lane 3, 124 bp) transcripts; C) cDNA sequence chromatograms of WT (left) and Mut (right) *MECP2_E1 *transcripts.** The *MECP2* exon 1–3 boundary sequence for the WT mRNA is indicated with a blue arrow, whereas *MECP2* exon 1–3 boundary sequence of the Mut mRNA is indicated with a red arrow. The 16bp sequence that is deleted in the Mut transcript between the cryptic splice junction and correct splice junction is indicated within the WT sequence using red brackets. **D**) Illustration of the predicted amino acid sequence of the MeCP2_E1 WT (green) and Mut (orange). The change of Glu at position 17 into Lys followed by a frameshift and introduction of a stop codon after 16 amino acids is indicated.

Here, we have identified a novel synonymous mutation in the coding portion of exon1 of *MECP2_E1* at position *c.48C>T*. This mutation was initially characterized as a silent mutation as it changes the 16^th^ codon from GGC to GGU/T, both of which code for glycine, i.e. *p.Gly16Gly*. *In silico* analysis of this synonymous variant predicts potential activation of a cryptic mRNA splice donor site upstream of the exon 1 splice donor site, which could result in a frameshift in the mRNA during protein translation and hence may ultimately result in protein truncation (Figure 2). Here, we present molecular evidence through analysis of mRNA sequence and mRNA quantization, confirming the activation of this cryptic splice donor.

## Materials and methods

### Patient ascertainment/assessment

The proband was ascertained and assessed through the Clinical Genetics Department at the Naval Medical Center San Diego, and recruited into a study of *MECP2* based at the Campbell Family Mental Health Research Institute, Centre for Addiction & Mental Health, Toronto. Institutional research ethics approval was obtained for this study, and written consent for the study was given by the proband’s parents. Recruitment of control subjects for mRNA quantification analysis has been described previously [16].

### Bioinformatic analysis

Potential splice sites were predicted using the online neural network tool at the Berkeley Drosophila Genome Project (BDGP) (http://www.fruitfly.org/seq_tools/splice.html), also using Human Splicing Finder (HSF; http://www.umd.be/HSF/) and MaxEntScan (genes.mit.edu/burgelab/ maxent/Xmaxentscan_scoreseq.html). ExPASy Swiss institute of bioinformatics (http://web.expasy.org/translate) online translate tool was used to identify the potential effect of this splice site mutation on the reading frame of the mutated version of protein.

### Cell culture and RNA preparation

Total RNA from the patient was extracted from a) lymphocytes drawn into Tempus™ tubes (Invitrogen Life Technologies), following the manufacturer’s protocol for PureLink® RNA Mini Kit (Invitrogen Life Technologies), and b) from Epstein-Barr Virus (EBV)-transformed blood cells (lymphoblasts), grown in RPMI medium supplemented with 15% Fetal Bovine Serum (FBS) and 1X penicillin streptomycin, with RNA extracted using Trizol method (all from Invitrogen Life Technologies).

### Reverse transcriptase polymerase chain reaction (RT-PCR) &**Sanger Sequencing**

RT-PCR analysis targeting the N-terminal coding regions for *MECP2_E1* and *MECP2_E2* was performed using sets of oligonucleotides designed with Primer Express 3.0 software (Applied Biosystems, Foster City, CA USA) (See Table 1). First strand cDNA was synthesized using Superscript III (Invitrogen) from RNA treated with DNase I (Fermentas). Following RT-PCR amplification across *MECP2* exons 1 to 3, sequencing analysis of the gel-eluted product (Qiagen) was performed at The Centre for Applied Genomics (http://www.tcag.ca) using gene specific primers (Figure 2).

**Table 1:** **RT-PCR assay details: PCR primer and probe sequences, or TaqMan™ assay ID**

**Assay specification**	**Assay ID**	**Assay details**
Detects Normal MeCP2_E1 transcript only	MeCP2_E1 NL	Left 5′-CGGAGGAGGAGGAGGA-3′
		Right 5′-GGAGGTCCTGGTCTTCTGACTT-3′
		Probe 6FAM-5′-AGGAGGAGAGACTGGAA3′
Detects Mutated MeCP2_E1 transcript only	MeCP2_E1 Mut	Left 5′-GTAAAAGCCGTCCGGAAAAT-3′
		Right 5′-TGCTTGCCCTCTTTCTCTTC-3′
		Probe 6FAM-5′-AGGAGGAGGGAAGAAA-3′
Detects MeCP2_E2 transcript only	MeCP2_E2	TaqMan™ Pre-Designed Gene expression Assay
		ID number: Hs00172845
Detects PGK1 transcript only (Endogenous control)	PGK1	TaqMan™ Pre-Designed Gene expression Assay
		ID number: Hs99999906

### TA cloning, colony screening and high resolution gel electrophoresis

Gel-eluted PCR product obtained from the previous step was cloned into the pDRIVE vector according to the manufacturer’s instruction (Qiagen). Selection of recombinant colonies was primarily done using α-complementation of the β-galactosidase gene with isopropyl β-D-1-thiogalactopyranoside (IPTG) supplemented LB agar. Colony-PCR of individually picked bacterial colonies was carried out using T7 and SP6 as forward and reverse primers. PCR-amplification with forward primer 5′-CCGAGCGGAGGAGGAGGAGG-3′ from exon 1 and reverse primer 5′-TGCTTGCCCTCTTTCTCTTC-3′ (exon 3) was followed by 2% high resolution agarose gel electrophoresis at 120V for 1 hour. PCR products were seen at the expected sizes, i.e. 140 bp for the normal transcript TA clone, and 124 bp for the mutated transcript TA clone, alongside 50 bp DNA ladder (Fermentas) (Figure 2).

### 5′ nuclease assay for relative mRNA quantification (qRT-PCR)

**Figure 3:**
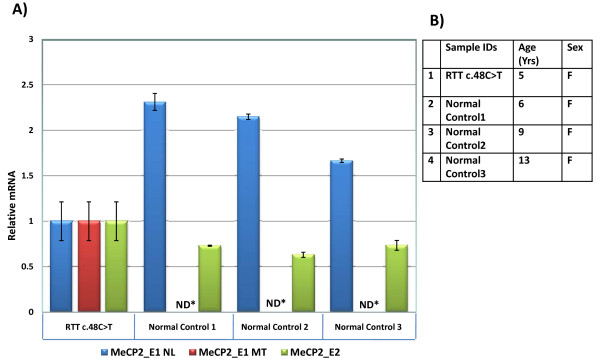
**5**′ **Nuclease Assay: A) Relative fold-change in the gene expression of *MECP2_E1 *normal (NL), *MECP2_E1 *mutant (MT), and *MECP2_E2 *transcripts in RTT individual with c.48C>T substitution, and three healthy control subjects.** All samples were normalized to endogenous control *PGK1*. Fold-change in the gene expression was calculated using ΔΔCt method and shown after log transformation. Error bars indicate standard error of the means. All reactions were done using three biological replicates per sample. **B**) Subject details. (ND*: Not detected).

Quantification of mutant and wild type transcripts was carried out using custom designed TaqMan® qRT-PCR gene expression assays (Figure 3B), using cDNA from the proband and three healthy female control individuals. Applied Biosystems® TaqMan® Universal PCR Master Mix, with optimal amplification conditions were used for all assays. All samples were analyzed in triplicate (three biological replicates) and normalized using phosphoglycerate kinase 1 gene (*PGK1)* as an endogenous control. All assays were designed according to the manufacturer’s instructions and the Primer Express 3.0 software tool (Applied Biosystems, Foster City, CA USA).

### Data analysis

Quantitative analyses of the data were carried out using ViiA™ 7 System SDS software (Applied Biosystems, Foster city USA). Greater than 36 Cycle threshold (Ct) value (from a total of 40 cycles of amplification) were considered as undetected [17]. Fold-differences in gene expression were calculated using the comparative cycle threshold method (ΔΔ Ct method) [17,18]. Statistical analysis was performed to calculate standard error of means (S.E.M) and standard deviations (SD) using Microsoft Excel tool for descriptive statistics.

## Results

The clinical details of the patient are reported here:

### ***Patient 1 information***

The patient is a five-year three-month-old female born of non-consanguineous parents. She has a younger brother who is developing normally. Her parents are both healthy, cognitively normal, without history of seizures. The patient was born at full term after an uncomplicated pregnancy. Birth weight was 50th centile, and head circumference, 25th centile. Concerns about her development arose around nine months of age when her progress seemed to slow, although without suggestion of regression. She sat at six months, walked at 18 months, began babbling at nine months of age but speech never progressed beyond that, and babbling has mostly disappeared. At two years of age she began having seizures. Cranial computed tomography (CT) and magnetic resonance imaging (MRI) at that time were normal. Electroencephalography (EEG) showed multifocal sharp waves. She developed multiple seizure types over time; tonic-clonic, complex partial, absence, and atonic seizures. Brain MRI was repeated at 4.5 years to include spectroscopy and was also normal.

In terms of the revised diagnostic criteria for RTT [19], she meets all four main criteria (loss of acquired purposeful hand skills, loss of acquired spoken language, gait abnormaility and sterotpical hand movelments) and at least seven out of 11 of the supportive criteria (breathing disturbance while awake, bruxism while awake, impaired sleep pattern, abnormal muscle tone, growth retardation, diminished response to pain and intense eye communication (“eye pointing”). Hands and feet were cold, although not small. Thus she meets criteria for typical or classic RTT in all except that regression is still ongoing, and a period of recovery or stabilization has not yet been established. Thus we have designated her with a diagnosis of typical Rett syndrome.

### Molecular and bioinformatics analyses

Metabolic evaluations and karyotyping were normal. Comparative genomic hybridization identified a 560 Kb (chr14: 37,627,931-38,187,142; hg19) duplication at 14q21.1 which contains two genes; mirror-image polydactyly gene 1 (*MIPOL1)* which is associated with mirror image polydactyly, and Forkhead box protein A1 (*FOXA1)* which is a transcription factor found to be overexpressed in some tumor types. This rare gain copy number variant was also present in the father’s DNA, thus excluding non-paternity. Molecular evaluations have also included normal Angelman syndrome methylation analysis, ubiquitin-protein ligase E3A (*UBE3A)* sequence analysis, and screening using a mitochondrial DNA point mutation panel.

*MECP2* sequence analysis identified a heterozygous single nucleotide substitution from C→T in *MECP2* exon 1 at nucleotide position 48 of the *MECP2_E1* coding sequence (*c.48C>T*)**.** This substitution was not present in either parent, and is likely to be *de novo*. The variant has not been reported in large scale whole exome or genome sequencing projects (1000 Genomes (phase 1 integrated release) and the NHLBI Exome Sequencing Project (ESP6500 data release)). The substitution is at the 3^rd^ (wobble) position of a glycine codon, and does not alter the coded amino acid (i.e. a synonymous change). According to the BDGP splice site prediction, the donor score for nucleotide positions c.47-48 is altered from <0.01 (WT) to 0.85 (Mutant; Figure 2A). Analysis using other algorithms supports this (Additional file 1: Table S1). The agarose gel electrophoresis of PCR amplified cDNA shows two bands for the *MECP2_E1* transcript for the affected individual. Upon separation of the mutated version from the normal transcript by cloning into a TA cloning vector, followed by sequencing analysis, it was revealed that the transcript resulting from *c.48C>T* lacks 16 nucleotides at the 3′end of exon 1 (*r.[=, 48_63del]*) (Figure 2B, C), indicating that *c.48C>T* results in a premature splicing event, as predicted. Although it splices with the correct acceptor site at intron 2/exon 3, due to the 16 nt deletion in exon 1 it is predicted to result in a frameshift event, changing glutamic acid (Glu) at position 17 into lysine (Lys), and leading to a truncated protein with a stop codon after 16 amino acids (p.Glu17Lysfs*16) (Figure 2D).

Analysis of the effect of *c.48C>T* on splicing for the *MECP2_E2* splice variant showed similar results, with the last 16 nt of exon 1 missing, but the correct usage of the splice acceptor at the intron 1/exon 2 boundary. As this deletion lies within 5′ untranslated region (UTR), it is not predicted to affect the 486 amino acid open reading frame of *MECP2_E2*, however, bioinformatic analysis shows that it results in an upstream open reading frame (ORF) 27 amino acids in length. This may putatively reduce the efficiency of translation of the full length ORF.

Further, quantification of the mutated and wild type mRNA transcripts showed a significant difference in the transcription levels. Approximately two-fold average decrease in expression of the WT *MECP2_E1* transcript (*MECP2_E1* NL) was observed in Patient 1, whereas mutated transcripts (*MECP2_E1* MT) were not detected (ND) in the normal samples. Relative expression of the *MECP2_E2* isoform was also quantified. An average of ~1.4-fold increase in the expression of the WT *MECP2_E2* transcripts was observed in Patient 1 (Figure 3A).

No mutant c.48C>T alleles from the patient showing the correct splicing at the canonical exon 1 splice donor site were identified in either *MECP2_E1* or *E2* transcripts, and all transcripts that did show splicing at the canonical exon 1 donor site were derived from the WT allele.

## Discussion

Synonymous mutations are generally considered as non-pathogenic, and are not expected to change the protein’s function. This paradigm has been challenged in recent years, with evidence that changes in codon usage may have consequences for the efficiency and speed of translation, that, in turn, may then affect protein folding and function [20,21]. In addition, the possible pathogenic mechanism of single nucleotide substitutions at codon wobble sites creating aberrant splice sites, is frequently overlooked, but while there are a number of reports with evidence for this at missense (non-synonymous) sites, there are fewer reports of such a mechanism in human disease involving synonymous changes [22]. To date, no synonymous mutation in *MECP2* has been proven to be pathogenic.

In this report, we have described the functional consequences of a synonymous mutation in *MECP2* exon 1, *c.48C>T* in a girl with a typical, albeit relatively mild form of RTT. This C>T transition changes the codon GGC into GGT, coding for the same amino acid i.e. glycine (*p.Gly16Gly*) (Figure 2A). At the transcriptional level this silent change clearly affects the gene expression by introducing a premature splice-donor site, resulting in the removal of 16nt of coding sequence from *MECP2_E1* transcripts *r.[=, 48_63del]*, This causes a frameshift, and premature truncation is predicted after 16 amino acid residues (KKSQKTSRTNPSSLKR*) (p.Glu17Lysfs*16) (Figure 2D). The effect of the mutation is similar for splice variant *MECP2_E2*, in that the last 16nt of exon 1 are removed, however, as this lies within the 5′UTR, there is no predicted effect on the protein. However, as the frame in the 5′UTR changes with the mutation and 16nt deletion, this may have an effect on the translation efficiency of *MECP2_E2*. The number and position of stop codons upstream to the translation start site remains unchanged, but while WT *MECP2_E2* has no apparent upstream ORF, the mutant form does have a 27 amino acid upstream ORF, which may compete for translational machinery with the correct ORF beginning in exon 2. Thus, while reduction in MeCP2_E1 protein levels is the most likely etiologically relevant consequence of the mutation in Patient 1, we cannot exclude that a reduction in MeCP2_E2 protein levels caused by translational interference is also contributing to the phenotype.

Reduced MeCP2 expression is likely one of the main pathogenic mechanisms in RTT [23]. Quantitative analysis of *MECP2_E1* and *E2* mRNA for Patient 1 suggest that this aberrant splice event is specific to the patient, and occurs in roughly half of the transcripts during post-transcriptional processing. A modest increase in *MECP2_E2* transcripts were observed in Patient 1 (Figure 3). It is possible that removal of the 16nt at the end of exon 1 may remove inhibitory sequences, permitting increased transcription of the *E2* mRNA, which could possibly be partially compensating for the loss of *MECP2_E1*, resulting in a milder RTT phenotype. Also, skewed X-inactivation may favor the WT allele in Patient 1, however information on X-inactivation was not available for this patient.

In summary, we have found a synonymous substitution in *MECP2* exon 1 coding region, which results in a splicing defect, which is predicted to lead to a truncated protein. We cannot, however, exclude the possibility that other mechanisms are also involved, for instance the possible effect on translation timing and efficiency (and thus protein folding and function) of the switch in codon usage at Gly16 from a high to low frequency codon [20,21], or a contributory effect of translational competition for the *MECP2_E2* splice variant from the mutated allele. We recommend the re-evaluation of all *de novo* synonymous substitutions in *MECP2*. In particular, through *in silico* analysis of all silent changes in *MECP2* reported on the Rettbase website and in the NHLBI ESP6500 exome sequence database, we found that the change c.627G>A (p.Val209Val) increases the donor splice site prediction score (Splice Site Prediction by Neural Network [24]) from 0.36 to 0.91 (out of 1.0), c.948C>G (Val316Val) changes the score from 0.09 to 0.42., and c.999G>T (Gly333Gly) changes the score from 0 to 0.59. These three substitutions should clearly be re-evaluated for possible *MECP2* mRNA splicing aberrations. Also, we recommend that algorithms used in the analysis of next generation sequence data be updated to predict the effect on splicing machinery at exonic sites away from the known splice donor and acceptor sites.

## Competing interests

The authors listed above declare that there are no conflicts of interest with the submitted work.

## Authors’ contributions

TIS designed and performed the molecular genetic studies, with support from KM and JBV. TIS assisted with interpretation of the data, prepared figures, tables and contributed to the manuscript draft. MJW ascertained the family and performed the clinical evaluations. JBV assisted with the study design and interpretation of the data, and prepared the final draft of the manuscript. All authors read and approved the final manuscript.

## Supplementary Material

Additional file 1: Table S1 *In silico* analysis of c.48C>T substitution for effect on donor splice site prediction using four different algorithms [25-27]. Click here for file

## References

[B1] Lewis JD Meehan RR Henzel WJ Maurer-Fogy I Jeppesen P Klein F Bird A Purification, sequence, and cellular localization of a novel chromosomal protein that binds to methylated DNA Cell 1992 69 6 905 914 10.1016/0092-8674(92)90610-O 1606614

[B2] Amir RE Van den Veyver IB Wan M Tran CQ Francke U Zoghbi HY Rett syndrome is caused by mutations in X-linked MECP2, encoding methyl-CpG-binding protein 2 Nature Genet 1999 23 2 185 188 10.1038/13810 10508514

[B3] Nan X Meehan RR Bird A Dissection of the methyl-CpG binding domain from the chromosomal protein MeCP2 Nucleic Acids Res 1993 21 21 4886 4892 10.1093/nar/21.21.4886 8177735 PMC311401

[B4] Nan X Campoy FJ Bird A MeCP2 Is a Transcriptional Repressor with Abundant Binding Sites in Genomic Chromatin Cell 1997 88 4 471 481 10.1016/S0092-8674(00)81887-5 9038338

[B5] Nan X Ng HH Johnson CA Laherty CD Turner BM Eisenman RN Bird A Transcriptional repression by the methyl-CpG-binding protein MeCP2 involves a histone deacetylase complex Nature 1998 393 6683 386 389 10.1038/30764 9620804

[B6] Nikitina T Ghosh RP Horowitz-Scherer RA Hansen JC Grigoryev SA Woodcock CL MeCP2-chromatin interactions include the formation of chromatosome-like structures and are altered in mutations causing Rett syndrome J Biol Chem 2007 282 38 28237 28245 10.1074/jbc.M704304200 17660293

[B7] Ghosh RP Horowitz-Scherer RA Nikitina T Shlyakhtenko LS Woodcock CL MeCP2 Binds Cooperatively to Its Substrate and Competes with Histone H1 for Chromatin Binding Sites Mol Cell Biol 2010 30 19 4656 4670 10.1128/MCB.00379-10 20679481 PMC2950531

[B8] Yang C van der Woerd MJ Muthurajan UM Hansen JC Luger K Biophysical analysis and small-angle X-ray scattering-derived structures of MeCP2–nucleosome complexes Nucleic Acids Res 2011 39 10 4122 4135 10.1093/nar/gkr005 21278419 PMC3105411

[B9] Mnatzakanian GN Lohi H Munteanu I Alfred SE Yamada T MacLeod PJ Jones JR Scherer SW Schanen NC Friez MJ Vincent JB Minassian BA A previously unidentified MECP2 open reading frame defines a new protein isoform relevant to Rett syndrome Nat Genet 2004 36 339 341 10.1038/ng1327 15034579

[B10] Kriaucionis S Bird A The major form of MeCP2 has a novel N terminus generated by alternative splicing Nucleic Acids Res 2004 32 5 1818 1823 10.1093/nar/gkh349 15034150 PMC390342

[B11] Harvey C Menon SD Stachowiak B Noor A Proctor A Mensah AK Mnatzakanian GN Alfred SE Guo R Scherer SW Kennedy JL Roberts W Srivastava AK Minassian BA Vincent JB Sequence Variants Within Exon 1 of *MECP2* Occur In Females With Mental Retardation Am J Med Genet Part B 2007 144 355 360 17171659 10.1002/ajmg.b.30425

[B12] Calfa G Percy AK Pozzo-Miller L Experimental models of Rett syndrome based on Mecp2 dysfunction Exp Biol Med 2011 236 1 3 19 10.1258/ebm.2010.010261 PMC3059199 21239731

[B13] Saunders CJ Minassian BE Chow EW Zhao W Vincent JB Novel exon 1 mutations in MECP2 implicate isoform MeCP2_e1 in classical Rett syndrome Am J Med Genet A 2009 149A 1019 1023 10.1002/ajmg.a.32776 19365833

[B14] Fichou Y Nectoux J Bahi-Buisson N Rosas-Vargas H Girard B Chelly J Bienvenu T The first missense mutation causing Rett syndrome specifically affecting the MeCP2_e1 isoform Neurogenet 2009 10 2 127 133 10.1007/s10048-008-0161-1 19034540

[B15] Itoh M Tahimic CG Ide S Otsuki A Sasaoka T Noguchi S Oshimura M Goto Y Kurimasa A Methyl CpG-binding protein isoform MeCP2_e2 is dispensable for Rett syndrome phenotypes but essential for embryo viability and placenta development J Biol Chem 2012 287 17 13859 13867 10.1074/jbc.M111.309864 22375006 PMC3340147

[B16] Gianakopoulos PJ Zhang Y Pencea N Orlic-Milacic M Mittal K Windpassinger C White SJ Kroisel PM Chow EW Saunders CJ Minassian BA Vincent JB Mutations in MECP2 exon 1 in classical Rett patients disrupt MECP2_e1 transcription, but not transcription of MECP2_e2 Am J Med Genet B Neuropsychiatr Genet 2012 159B 2 210 216 10.1002/ajmg.b.32015 22213695

[B17] Sheikh TI Qadri I Expression of EBV Encoded viral RNA 1, 2 and anti-inflammatory Cytokine (interleukin-10) in FFPE lymphoma specimens: a preliminary study for diagnostic implication in Pakistan Diag Pathol 2011 6 1 8 10.1186/1746-1596-6-1 PMC3157411 21791113

[B18] Livak KJ Schmittgen TD Analysis of Relative Gene Expression Data Using Real-Time Quantitative PCR and the 2^-ΔΔ*C*T^ Method Methods 2001 25 4 402 408 10.1006/meth.2001.1262 11846609

[B19] Neul JL Kaufmann WE Glaze DG Christodoulou J Clarke AJ Bahi-Buisson N Leonard H Bailey ME Schanen NC Zappella M Renieri A Huppke P Percy AK; **RettSearch Consortium** Rett syndrome: revised diagnostic criteria and nomenclature. Ann Neurol 2010 68 6 944 950 10.1002/ana.22124 PMC3058521 21154482

[B20] Kimchi-Sarfaty C Oh JM Kim IW Sauna ZE Calcagno AM Ambudkar SV Gottesman MM A "silent" polymorphism in the MDR1 gene changes substrate specificity Science 2007 315 5811 525 528 10.1126/science.1135308 17185560

[B21] Edwards NC Hing ZA Perry A Blaisdell A Kopelman DB Fathke R Plum W Newell J Allen CE SG Shapiro A Okunji C Kosti I Shomron N Grigoryan V Przytycka TM Sauna ZE Salari R Mandel-Gutfreund Y Komar AA Kimchi-Sarfaty C Mandel-Gutfreund Y Komar AA Kimchi-Sarfaty C Characterization of coding synonymous and non-synonymous variants in ADAMTS13 using ex vivo and in silico approaches PLoS One 2012 7 6 e38864 10.1371/journal.pone.0038864 22768050 PMC3387200

[B22] Cartegni L Chew SL Krainer AR Listening to silence and understanding nonsense: exonic mutations that affect splicing Nat Rev Genet 2002 3 285 298 10.1038/nrg775 11967553

[B23] Squillaroa T Alessioc N Cipollaroa M Melone MA Hayek G Renieri A Giordano A Galderisi U Reduced expression of *MECP2* affects cell commitment and maintenance in neurons by triggering senescence: new perspective for Rett syndrome Mol Biol Cell 2012 23 8 1435 1445 10.1091/mbc.E11-09-0784 22357617 PMC3327309

[B24] Reese MG Eeckman FH Kulp D Haussler D Improved Splice Site Detection in Genie J Comp Biol 1997 4 3 311 323 10.1089/cmb.1997.4.311 9278062

[B25] Desmet FO Hamroun D Lalande M Collod-Beroud G Claustres M Beroud C Human Splicing Finder: an online bioinformatics tool to predict splicing signals Nucleic Acid Research 2009 37 1 14 10.1093/nar/gkn923 PMC2685110 19339519

[B26] Dogan RI Getoor LM Wilbur WJ Mount SM SplicePort—An interactive splice-site analysis tool Nucleic Acids Research 2007 35 W285 W291 10.1093/nar/gkm407 17576680 PMC1933122

[B27] Yeo G Burge CB Maximum entropy modeling of short sequence motifs with applications to RNA splicing signals J Comput Biol 2004 11 2–3 377 394 15285897 10.1089/1066527041410418

