# Supplementary material for: A synonymous change, p.Gly16Gly in MECP2 Exon 1, causes a cryptic splice event in a Rett syndrome patient
Source: Orphanet J Rare Dis. 2013 Jul 19;8:108. doi: 10.1186/1750-1172-8-108 (PMC3729535; doi:10.1186/1750-1172-8-108)
Supplement: Additional file 1: Table S1 — In silico analysis of c.48C>T substitution for effect on donor splice site prediction using four different algorithms [25-27]. [file 1750-1172-8-108-S1.doc]

**Supplementary Materials**

**Supplementary Table 1: *In silico* analysis of c.48**C>T substitution for effect on donor splice site prediction using four different algorithms.****

| **Srl. No.** | **Tool** | **Splice Site prediction (Mutant)** | **Splice Site prediction (Wild type)** | **Reference** |
| --- | --- | --- | --- | --- |
| 1. 3 | ****Human Splicing finder (Inserum)**** [**http://www.umd.be/HSF/**](http://www.umd.be/HSF/) | | Splice | Short sequence | Consensus value (0-100) | | --- | --- | --- | | D* | gag**gt**gagg | 95.15 | | | Splice | Short sequence | Consensus value (0-100) | | --- | --- | --- | | D* | gag**gc**gagg | 68.31 | | [25] |
| 1. 1 | SplicePort  http://spliceport.cbcb.umd.edu/ | | Slice | Short sequence | Score threshold (-10) | | --- | --- | --- | | D* | aggag**gt**gagga | 0.415038 | | D* | agact**gt**gagtg | 0.0927092 | | | Splice | Short sequence | Score threshold (-10) | | --- | --- | --- | | A** | ggaggag**gc**gag | -5.517 | | D* | agact**gt**gagtg | 0.476584 | | [26] |
|  | ****Human Splicing Finder (Inserum)**** [**http://www.umd.be/HSF/**](http://www.umd.be/HSF/) | | Splice | 5’ Motif | 5’ Score | | --- | --- | --- | | D* | gag**gt**gagg | 8.41 | | | Splice | 5’ Motif | 5’ Score | | --- | --- | --- | | D* | gag**gc**gagg | 0.65 | | [27] |
| 1. 2 | ****Splice Site Prediction by Neural Network**** http://www.fruitfly.org/seq_tools/splice.html | | Splice | Short sequence | Score Cutoff 0-1 | | --- | --- | --- | | D* | ggaggag**gt**gaggag | 0.85 | | | Splice | Short sequence | Score Cutoff 0-1 | | --- | --- | --- | | D* | ggaggag**gc**gaggag | <0.01 | | [23] |

* Splice Donor site

** Splice Acceptor Site
